# Predictors of HIV Viral Load Suppression After Enhanced Adherence Counseling, Nekemte, Ethiopia

**DOI:** 10.1007/s44197-024-00246-0

**Published:** 2024-05-22

**Authors:** Yohannis Mosisa, Adisu Ewunetu, Kitesa Biresa Duftu, Bayise Biru, Debelo Diriba, Adisu Tafari Shama, Melese Lemi, Getahun Fetensa, Bikila Regassa Feyisa

**Affiliations:** 1 Wallaga University Comprehensive Specialized Hospital, Nekemte, Ethiopia; 2 Department of Public Health, Institute of Health Sciences, Wallaga University, Nekemte, Ethiopia; 3 Department of Epidemiology, Faculty of Public Health, Institute of Health, Jimma University, Nekemte, Ethiopia; 4 Department of Human Nutrition and Dietetics, Faculty of Public Health, Jimma University, Nekemte, Ethiopia; 5 Food for the Hungry Ethiopia, Western Region, Nekemte, Ethiopia; 6 Early Warning and Preparedness Case Team, Public Health Emergency Management and Health Research Directorate, Oromia Health Bureau, Oromia, Ethiopia; 7 Department of Nursing, School of Nursing and Midwifery, Institute of Health Sciences, Wallaga University, Nekemte, Ethiopia; 8 Department of Health Behavior and Societies, Faculty of Public Health, Institute of Health, Jimma University, Jimma, Ethiopia

**Keywords:** Viral load suppression, Enhanced adherence counseling, Public health facilities, Nekemte town

## Abstract

**Background:**

Enhanced adherence counseling refers to the counseling intervention for Human Immunodeficiency Virus (HIV) patients with an elevated viral load result, a viral load of > 1000 copies/ml, on a routine or need-based viral load test. The Federal Ministry of Health, Ethiopia, has launched routine viral load testing and enhanced adherence counseling since 2016 for high-viral load people living with HIV, which is applicable throughout the country for all health facilities providing HIV care and treatment. Our study aimed to assess viral load suppression after enhanced adherence counseling and its predictors among high viral load people living with HIV who were on antiretroviral therapy.

**Method:**

We conducted a health facility-based retrospective follow-up study among 352 HIV-infected high-viral load people enrolled in enhanced adherence counseling from July 2018 to June 2021 in Nekemte town public health facilities. Cox proportional hazard analysis was used to identify independent predictors.

**Results:**

The overall 65.1% of 352 persons on antiretroviral treatment achieved HIV viral load suppression after enhanced adherence counseling, (15.01 per 100 person months (95% CI13.02-16.99)). The median time to viral load suppression was 5 months. Age ≥ 15 years (AHR = 1.99, 95% CI: 1.11–3.57), no history of opportunistic infection (AHR = 2.01, 95% CI: 1.18–3.41), and not using substances (AHR = 2.48, 95% CI: 1.19–5.14) were more likely to have viral load suppressed, while having an initial viral load count greater than 50,000 RNA copies/ml (AHR = 0.56, 95% CI: 0.37–0.85) were less likely to have viral load suppressed after enhanced adherence counseling.

**Conclusion:**

Age, history of opportunistic infections, substance use, and an initial viral load count > 50,000 RNA copies/mL were significant predictors of viral load suppression. Enrolling all high-viral-load patients in enhanced adherence counseling is recommended for viral load suppression.

## Introduction

Monitoring individuals receiving Antiretroval Therapy (ART) is important to ensure successful treatment, identify adherence problems, and determine whether and which ART regimens should be switched in cases of HIV treatment failure [1, 2]. Periodic viral load testing is the gold standard for HIV treatment monitoring compared to immunologic or clinical methods [3].

Enhanced Adherence Counseling (EAC) refers to the counseling intervention for HIV patients with an elevated viral load result, a viral load of > 1000 copies/ml on a routine or need-based viral load test. A package for EAC consists of one to six sessions, which may be given on a monthly basis. The objective of these sessions is to assess the barriers to adherence and design strategies to overcome these barriers. The first of these sessions is given on the day the high viral load result is given to the patient, with subsequent sessions following monthly drug refill intervals [4].

The Federal Ministry of Health, Ethiopia, has launched routine viral load testing and EAC since 2016 for high viral load People Living With HIV (PLWHIV), which is applicable throughout the country for all health facilities providing HIV care and treatment. According to the World Health Organization (WHO) estimation, up to 70% of patients with an initial high viral load count achieved virologic suppression after the EAC session [5], but only 66% of patients in Ethiopia achieved it [6]. Surveys indicate that sociodemographic factors, viral load count at the start of the EAC session, and duration on ART were some of the predictors of viral load suppression after EAC [6].

In Ethiopia, a few studies conducted in west Gojjam [7] and North Wollo [8] found that the proportion of viral load suppression was 51.7% and 66%, respectively, following the EAC. These findings indicated that viral suppression following EAC is below the standard when compared with WHO recommendations. Ethiopia is a very broad country with many socio-economic problems, and hence such studies need to be exhaustively conducted throughout the country. Besides, the eastern part of Ethiopia was dealing with multiple emergencies such as internal displacement, the outbreak of COVID-19, malaria, and measles, which may affect the viral load suppression after EAC. To this end, this study was designed to assess viral load suppression after EAC and its predictors among high viral load PLWHIV in public health facilities in Nekemte town, in western Ethiopia.

## Methods and Materials

### Study Setting, Design, and Period

We conducted a retrospective analysis of routine HIV care data from public health facilities in Nekemte Town, the largest town in Western Ethiopia, from April 15 to June 15, 2022. The study was conducted in four public health facilities (one specialized hospital, one referral hospital, and two health centers) found in Nekemte town. All of the health facilities conduct routine viral load testing for all HIV-infected people and provide EAC services for high-viral-load patients as per national guidelines. During the data collection, 3,366 PLWHIV were taking ART in the four health facilities.

### Study Population

All HIV-infected people who had a viral load > 1000 copies/mL after 6 months on ART from July 2018 to June 2021 in the study facilities.

### Sample Size and Sampling Procedures

**Fig. 1:**
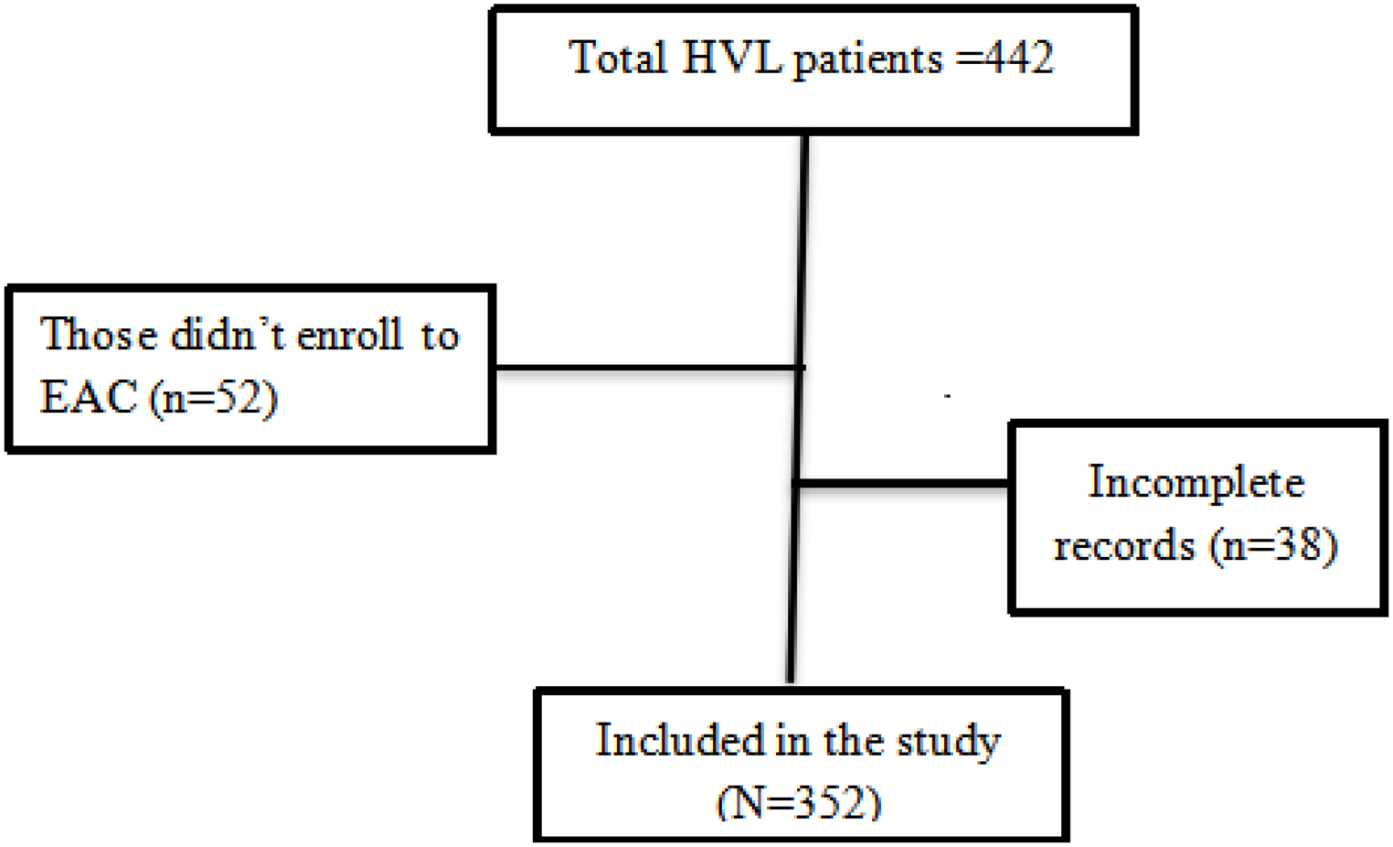
Schematic diagram showing sampling procedure for viral load suppression after enhanced adherance counseling among high viral load people living with HIV who were on antiretroviral therapy from July 2018 to June 2021 in Nekemte town public health facilities, Nekemte, Ethiopia, 2022

We considered all patients with a high viral load enrolled in EAC and who were on ART from July 2018 to June 2021 from each public health facility in the town as a final sample size. The study participants were taken from the viral load registration book (Fig. 1).

- **Dependent variable**: time to viral load suppression after EAC.
- **Independent variables**.
- **Socio-demographic factors**: Sex, Age, Weight, Marital Status, Educational Status, Residence, and Occupation.
- **Clinical and laboratory factors**: functional status of the patient, WHO clinical stage, viral load count at the start of the EAC session, history of hospitalization, time to start the EAC, duration on ART, past opportunistic infections.
- **Nutritional and behavioural factors**: nutritional status, level of ART adherence, substance use (alcohol or Khat intake).
- **ART and treatment-related factors**: therapeutic regimen, isoniazid preventive therapy status, and cotrimoxazole preventive therapy status.

### Data Collection

For data abstraction, we developed a check list containing socio-demographic, nutritional, behavioural, clinical, laboratory, ART, and treatment-related variables. Six trained BSc nurses and one supervisor were recruited for data collection. High-viral-load patients were identified from viral load registration and retrieved by using medical record numbers. Data was extracted from the intake form, EAC sheet, ART follow-up form, laboratory request form, and high viral load registration book. If clinical parameters and laboratory results (CD4 count and WHO clinical stage) are not found at the start of EAC sessions, the data that is most recent to the starting date of EAC sessions is considered baseline data.

### Data Analysis

After coding and clearing, we entered the data into Epi Data version 3.1 and analyzed it using STATA version 14. Descriptive statistics were computed using frequencies and summary statistics, such as means, medians, and standard deviation. We used a life table to estimate the cumulative viral load suppression and the Kaplan-Meier curve (survival function) to present the median time to viral load suppression and estimate survival probability. To compare survival probability between different variables and identify whether there is a significant difference in survival probability between different covariates, a log-rank test was used. Bivariable Cox proportional regression was run to identify predictors of viral suppression after EAC, and variables with *P* < 0.25 were entered into a multivariable Cox proportional model. Before fitting the predictors into a multivariable model, all the proportionality assumptions were checked by a global test based on Schoenfeld residuals (Supplementary Table 1) and by examining log-log plots (Supplementary Table 2). The overall goodness of model fitness was checked by the Cox-Snell residual (Supplementary Table 3). Multi-collinearity was checked using the variance inflation factor. On the multivariable Cox proportional hazard model, Adjusted Hazard Ratio (AHR) with 95% confidence intervals and *P* < 0.05 was considered statistically significant. Finally, results were compiled and presented using tables, graphs, and texts.

### Ethical statement

The study was approved by the Wallaga University Research Ethics Review Committee (WURERC) (ref no: WU/RD/552/2014). An official letter was sent to the administrative office of the health facilities in the town. The need for informed consent was waived by WURERC since the study is a retrospective chart review. We performed all procedures in accordance with the relevant guidelines and reported this work following the preferred standard for reporting observational studies in epidemiology (STROBE) (Supplementary 1).

## Results

### Baseline Socio-Demographic Characteristics

The mean age of the study participants was 33.26 (± 12.09 SD), and 191 (54.3%) of them were female. 154 (43.7%) of study participants attained the primary level of education, and the majority of the participants, 323 (91.8%), live in urban areas. Nearly half (170, or 48.3%) of the clients were married (Table 1).

**Table 1:** Sociodemographic characteristics of high-viral-load people living with HIV enrolled in enhanced adherance counselling from July 2018 to June 2021 at nekemte town public health, Nekemte, Ethiopia, 2022

Covariates	Group	Final status of Viral load after EAC (n)		Total n*o* (%)
		Viral load suppressed	Censored	
Sex of the client	Male	96	65	161(45.7%)
	Female	133	58	191(54.3%)
Age of HVL clients	< 15 years	16	10	26(7.4%)
	≥ 15years	213	113	326(92.6%)
religion of the client	Orthodox	93	48	141(40.05%)
	Catholic	2	1	3(0.85%)
	Protestant	108	60	168(47.7%
	Muslim	26	14	40(11.4%
Educational status	Cannot read and write	46	15	61(17.3%)
	Primary	100	54	154(43.7%)
	Secondary	64	43	107(30.4%)
	Tertiary and above	19	11	30(8.5%)
Residence	Urban	209	114	323(91.8%)
	Rural	20	9	29(8.2%)
Marital status	Married	111	59	170(48.3%)
	Single	62	36	98(27.84%)
	Widowed	32	11	43(12.22%)
	Divorced	24	17	41(11.65%
Occupation	Housewife	44	16	60(17.05%)
	Farmer	14	8	22(6.25%)
	Merchant	41	23	64(18.18%)
	Government employed	25	11	36(10.23%)
	Students	26	17	43(12.21%)
	Daily laborer	28	25	53(15.06%
	Jobless	16	13	29(8.24%)
	Others	35	10	45(12.78%)

### Viral Load Suppression Status after EAC

**Fig. 2:**
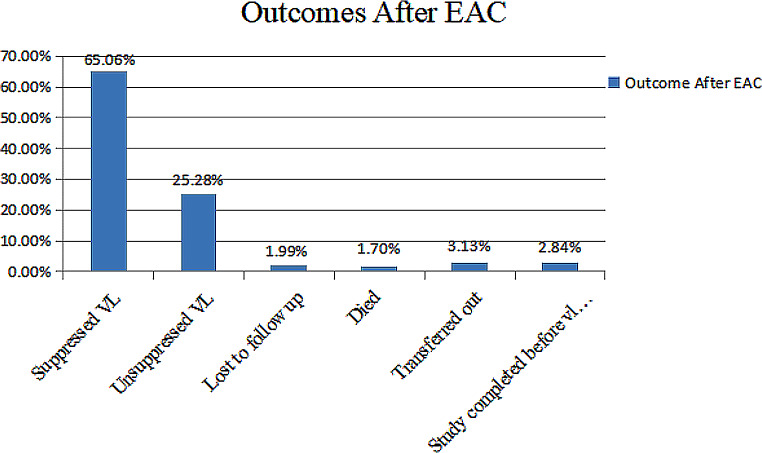
Q1

From the total of 352 high viral load cohorts of clients enrolled in EAC, about 229 (65.1%) achieved viral load re-suppression, whereas 7 (2%) were lost to follow-up, 6 (1.7%) died, and 11 (3.1%) were transferred to other facilities (Fig. 2).

After starting the EAC session, participants were followed for different periods, a minimum of 3 months and a maximum of 6 months, and contributed to 1525 person months of follow-up. The overall viral suppression rate of the cohort was found to be 15.01 (95% CI: 13.02–16.99) per 100 person-months of observation. The cumulative probability of survival at the end of the 3rd, 4th, 5th, and 6th months was 70.34%, 55.18%, 45.63%, and 12.63%, respectively. Among 229 virally suppressed clients after EAC sessions, 64 (28%) were virally suppressed after the completion of a 6-month EAC session, 25 (11%) in 5 months, 43 (18.77%) in 4 months, and 97 (42.35%) in 3 months. The highest viral load suppression rate was achieved after the completion of a 3-month EAC session, which was 21.9 per 100-person month, followed by 19.9, 13.16, and 9.4 per 100-person month at 4, 5, and 6 months, respectively. Table 2 describes it well.

**Table 2:** Cumulative survival of a high viral load people living with HIV who were enrolled in enhanced adherence counseling from July 2018 to June 2021 in nekemte town public health facilities, Nekemte, Ethiopia, 2022

Interval in months	Number of study participant	Viral load suppressed	Censored	Survival	Std. error	95% CI
3–4	352	97	50	0.7034	0.0253	0.6506–0.7497
4–5	205	43	11	0.5518	0.0285	0.4941–0.6056
5–6	151	25	13	0.4563	0.0293	0.3981–0.5125
6–7	113	64	49	0.1263	0.0232	0.0853–0.1757

### Time to Viral Load Suppression and Comparison of Survival Probability Among Categories of Covariates

**Fig. 3:**
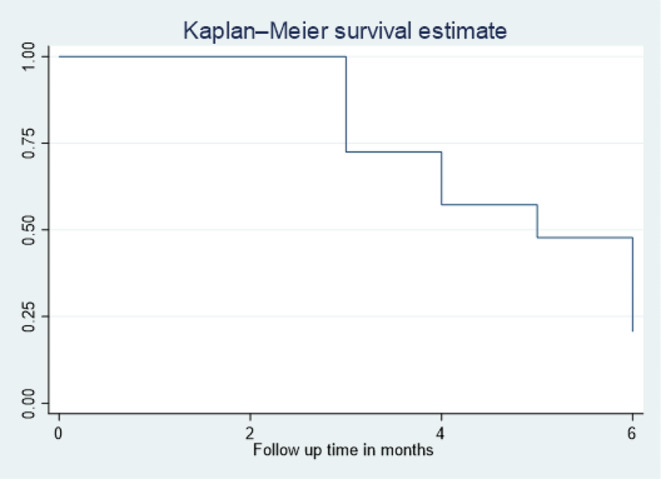
Overall Kaplan Meier survival estimate for patients enrolled in enhanced adherance counseling showing median time to viral load suppression in Nekemte town public health facilities, Nekemte, Ethiopia, 2022

The findings of our study indicate that the median time to complete an EAC session and suppress viral load was 5 months (Fig. 3). Clients aged 15 years and older (log-rank p value = 0.009), who were receiving the 1st line ART regimen (log-rank p-value = 0.029), who were on ART for > 60 months (log-rank p-value = 0.034), who had an initial viral load ≥ 50,000 RNA copies/mL (log-rank p-value < 0.001), with past opportunistic infections (log-rank p-value < 0.001), and those who did not use substances (log-rank p-value < 0.001) had a higher probability of viral load suppression rate when compared with their counterparts, respectively. On the other hand, the p-value of the log-rank test did not show a significant difference in survival probability among variables like sex, residence, WHO stage, Isoniazid treatment status, and nutritional status.

### Predictors of Viral Load Re-Suppression

When variables with a P-value < 0.25 on bivariable analysis were all included in the multivariable Cox proportional hazard model, age of the client, past opportunistic infections, initial viral load, and history of substance use showed statistically significant associations with viral load suppression (p-value < 0.05) (Table 3).

Clients aged 15 years and older were about 2 times more likely to have viral load suppression after EAC when compared to clients aged 15 years and younger (AHR = 1.99, 95% CI: 1.11–3.57). Participants who had no history of opportunistic infection were about 2 times more likely to have a viral load suppression rate compared to their counterparts (AHR = 2.01, 95% CI: 1.18–3.41). Participants with an initial viral load count > 50,000 copies/ml had lower viral load suppression after EAC by 44% when compared to those who had an initial viral load ≤ 5000 copies/ml (AHR = 0.56, 95% CI: 0.37, 0.85). Participants who do not use substances were 2.4 times more likely to have viral load suppression after EAC when compared with substance users (AHR = 2.48, 95% CI: 1.19–5.15).

**Table 3:** Multivariable analysis of predictors of viral load suppression after enhanced adherance counseling among high viral load people Living with HIV in public health facilities of Nekemte Town from July 2018 to June 2021, Nekemte, Ethiopia, 2022, (*n* = 352)

Covariates	Categories	Viral load status after EAC (n)		Crude hazard ratio with 95%CI	Adjusted hazard ratio with 95% CI
		Suppressed	Censored		
Sex of the client	Male	96	65	1	1
	Female	133	58	1.21(0.93, 1.57)	0.90 (0.68, 1.20)
Age	< 15 years	16	10	1	1
	≥ 15years	213	113	1.70(1.02, 2.84)	1.99(1.11, 3.57)*
Marital status	Married	111	59	1	1
	Single	62	36	0.82(0.60, 1.12)	1.00 (0.71, 1.42)
	Widowed	32	11	1.24(0.84, 1.84)	1.16(0.77, 1.76)
	Divorced	24	17	0.84(0.54, 1.31)	0.82 (0.52, 1.29)
Therapeutic regimen	1st line	217	112	1	1
	2nd line	12	11	0.66 (0.37, 1.18)	0.77 (0.42, 1.41)
Duration on ART in months	≤ 60	118	56	1.25( 0.96, 1.61)	1.24(0.95, 1.63)
	> 60	111	67	1	1
History past OI	Yes	31	63	1	1
	No	198	60	2.27(1.61, 3.44)	2.01 (1.18, 3.41)*
History of Hospitalization	Yes	15	24	1	1
	No	214	99	1.90 (1.13, 3.21)	0.76 (0.38, 1.55)
Cotrimoxazole preventive Therapy status	Treatment taken	57	29	0.85 (0.60, 1.19)	1.13 (0.78, 1.63
	On treatment	94	74	0.69 (0.52 0.94	0.92(0.62, 1.35)
	Not eligible	78	20	1	1
Initial viral load result	≤ 5000	94	20	1	1
	5001–49,999	101	53	0.72 (0.54, 0.95)	0.77 (0.57, 1.02)
	≥ 50,000	34	50	0.46(0.31, 0.69)	0.56 (0.37, 0.85)***
Substance use	Yes	8	27	1	1
	No	221	96	3.12(1.54, 6.33)	2.48(1.19, 5.15)**
Baseline CD4 (cells/ml)	< 350	85	84	1	1
	≥ 350	144	39	1.41(1.08, 1.84)	1.13 (0.79, 1.62

*Keys* *p < 0.05, **p < 0.01, ***p < 0.001; 1 is the reference category

## Discussion

The study indicates that the degree of viral re-suppression following EAC is almost at the WHO target [2] and is comparable to a study carried out in the North Wollo Zone in Ethiopia [6]. It is nevertheless far higher than the viral load suppression shown in Uganda [9] and Zimbabwe [8]. The discrepancy could be the result of variations in the study period and study environment. For example, the research from Zimbabwe was limited to hospital settings, while that from Uganda included only kids and teenagers. Besides the degree of education and counseling experience of medical professionals at various hospitals and clinics, on counseling also matters.

Comparably, this study’s rate of viral load suppression is higher than that of the study carried out in Ethiopia’s West Gojjam zone [7]. The reason for the discrepancy could be because this study was conducted after a different viremia clinic with medical professionals specially prepared to handle patients with high viral loads in facilities with high caseloads opened. Most importantly, the study’s findings supported the WHO’s recommendations, which specify that EAC should suppress the first high viral load (> 1000 copies/ml) with recurrent viral load measurements before modifying the treatment plan [4].

The study showed that the median time to complete the EAC session and viral load suppression was 5 months, which is comparable to the study conducted in the West Gojjam Zone [7]. The similarity may be due to similar methodology and follow-up periods of 6 months in both studies, but higher in the north Wollo zone, which may be due to a different methodology and follow-up duration in which they were followed up to 60 weeks after the initiation of EAC [6].

This study revealed that clients aged 15 years and older had more likely achieved viral load suppression compared to those aged 15 years and younger. It is consistent with studies conducted in Swaziland [10] and other studies around the world. This similarity may possibly be explained by their maturity, so that they relatively give better care for their health than children. Besides, the formulation of ART drugs for children may matter because they are not fixed-dose combinations, which may contribute to poor adherence. Another possible reason could be that children and adolescents undergo psychosocial stressors that are often unnoticed by adults and caregivers [11, 12].

Clients with an initial viral load count of ≥ 50,000 RNA copies/mL were 44% less likely to be virally suppressed after EAC as compared to those with an initial viral load of ≤ 5000 RNA copies/mL. This finding was supported by a study conducted in Zimbabwe and the north Wollo zone, Ethiopia [6, 8]. This may be due to the fact that increased viral loads are easily multiplying and becoming resistant to ART drugs, and clients develop different opportunistic infections [12].Clients who had no history of opportunistic infections were more likely to be virally suppressed when compared to their counterparts. This finding was supported by studies conducted in west Gojjam, Ethiopia, South Africa, Uganda, and north-eastern Ethiopia [7, 13–15]. It may be due to the fact that clients with opportunistic infections are most of the time immune-compromised individuals, and when there is immune decline, there is a higher rate of viral replication compared to individuals with intact immunity, which sustains the vicious cycle of immunity and viral replication [16].

The history of substance use by the study participants was a statistically significant predictor of viral load suppression. Similarly, when compared to non-drinkers in Florida and South Africa, excessive alcohol intake was linked to suboptimal ART adherence and HIV viral suppression [17, 18]. Alcohol use may impact HIV viral suppression via biological (immune dysfunction) and behavioural (ART adherence) pathways [19].

The findings of our survey indicate that clients’s sex, marital status, educational status, functional status, WHO stage, duration on ART, and ART regimen were not significant predictors of viral load suppression, which is similar to studies done in the Amhara region [7].

The users of these findings need to consider the following issues: Because the data were collected through documentary review, the analysis and interpretation of the data were restricted to only those variables that are available in the patient records. Some of the important variables, such as knowledge and awareness of clients’ HIV viral load, income, distance to health institutions, and history of their current sexual partner, which could have played a role in adherence and viral load suppression, were not assessed. Some variables, like substance abuse, were taken as they appeared in the EAC tool, which makes it difficult to measure the extent and duration of the client’s use of alcohol or Khat and restricts the analysis to only determining whether the patient is taking it or not.

In summary, nearly two-thirds of persons with HIV in the public health facilities in Nekemte town, Ethiopia, achieved viral load suppression after EAC. Besides, a significant association was observed between the age of the client, past opportunistic infections, initial viral load, and history of substance use.

Health institutions and professionals should prioritize enrolling all HIV patients with high viral loads into treatment programs, ensuring they receive proper care and support. Additionally, there should be a concerted effort to focus on children affected by HIV, with caregivers receiving counseling on the importance of adhering to the ART protocol outlined in the HIV care package for children. Patients who are also substance users should be encouraged to discontinue substance use and prioritize adherence to their ART regimens for optimal health outcomes. For researchers, it is essential to conduct prospective cohort studies to identify significant predictors of viral load suppression that may not have been detected in secondary data analysis, thereby advancing our understanding of HIV treatment efficacy and informing future interventions.

## Data Availability

The datasets generated and/or analyzed during the current study are available from the corresponding author on reasonable request.

## Abbreviations

AHR: Adjusted Hazard Ratio
AIDS: Acquired Immune Deficiency Syndrome
ART: Antiretroviral Therapy
CHR: Crude Hazard Ratio
EAC: Enhanced Adherence Counselling
EPHIA: Ethiopian Population-Based HIV Impact Assessment
HAART: Highly Active Antiretroviral Therapy
HIV: Human Immune Deficiency Virus
UNAIDS: United Nations-Acquired Immune Deficiency Syndrome
WHO: World Health Organization
WURH: Wallaga University Referral Hospital
